# Exploring Biomass Precursor Synthesis as a Determinant in Microbial Adaptation to Unadapted Carbon Sources with AdaptUC

**DOI:** 10.34133/research.0881

**Published:** 2025-10-17

**Authors:** Jingyi Cai, Jiayu Liu, Fan Wei, Wenjun Wu, Wenqi Xu, Yu Wang, Qianqian Yuan, Hongwu Ma

**Affiliations:** ^1^ Key Laboratory of Engineering Biology for Low-Carbon Manufacturing, Tianjin Institute of Industrial Biotechnology, Chinese Academy of Sciences, Tianjin 300308, China.; ^2^ National Center of Technology Innovation for Synthetic Biology, Tianjin 300308, China.; ^3^ University of Chinese Academy of Sciences, Beijing 101408, China.; ^4^ Tianjin University of Science & Technology, Tianjin 300457, China.

## Abstract

Industrial microorganisms often struggle to utilize renewable substrates such as methanol, formate, and xylose. Here, we introduce AdaptUC, a computational framework that demonstrates how the fraction of biomass precursors synthesized from unadapted carbon sources governs both the evolutionary driving force and the minimal substrate requirement. AdaptUC predicts gene knockout strategies for constructing the starting strain for adaptive laboratory evolution by selectively blocking metabolic pathways, thereby rendering specific precursor pools dependent on the unadapted substrate. We show that smaller dependency fractions correspond to higher driving forces for evolution of the starting strain. Case studies in *Escherichia coli* and *Corynebacterium glutamicum*, validated against experimental records and literature, confirm AdaptUC’s ability to identify knockout combinations that fine-tune precursor dependency and accelerate adaptation. By leveraging genome-scale metabolic models, AdaptUC navigates vast candidate pools without combinatorial explosion, reducing experimental screening and prioritizing strains with stronger evolutionary drives.

## Introduction

Industrial microorganisms are essential for sustainable biosynthesis processes, but they often struggle to utilize renewable substrates such as methanol, formate, and xylose. These substrates derived from nonfood materials or industrial waste like methanol (oxidized from methane or synthesized from carbon dioxide [1]), xylose (from agricultural waste), and formate (electrochemically synthesized from carbon dioxide [2]) hold great promise for industrial biosynthesis. However, many industrial strains cannot naturally assimilate these unadapted carbon sources due to factors such as the absence of assimilation pathways, redox imbalance [3], unfavorable enzyme kinetics [4], inadequate enzyme expression levels, or regulatory mechanisms [5]. Ultimately, this lack of adaptation is likely because these substrates were not present in the microorganisms’ natural environments during evolution [6]. An immediate solution is to introduce heterologous genes encoding the necessary pathways into the host cell, which has been effective in enabling *Corynebacterium glutamicum* to assimilate xylose [7] and *Escherichia coli* to grow on serine [8]. Nevertheless, a complete assimilation pathway may not suffice for growth on an unadapted carbon source. For example, the toxicity of intermediate formaldehyde still prevents unevolved *E. coli* from growing when a heterologous methanol utilization pathway is inserted [9,10]. Similarly, *E. coli* cannot utilize glycerol under anaerobic conditions due to redox imbalance and thermodynamic barriers [3], and *E. coli* grows poorly [11] on formate as a sole carbon source without adaptive laboratory evolution (ALE) [12,13].

To overcome these challenges, it is crucial to understand the factors determining the evolutionary driving force and minimal substrate requirements for the assimilation of unadapted carbon sources. ALE is a powerful technique that helps cells adapt and fine-tune their cellular machinery for better growth on new substrates. Given the starting strain’s difficulty in assimilating unadapted carbon sources, favored cosubstrates are required to support its growth initially. This is followed by gradually changing environmental conditions to force the strain to evolve toward a phenotype with high utilization capacity for the previously unadapted substrate.

A question in this context is how the proportion of biomass precursors that must be synthesized from unadapted carbon sources affects the performance of engineered starting strain for ALE. This question is important because if the proportion of biomass precursors from unadapted substrates plays a major role, it could serve as a guiding principle in designing new microbial strains capable of efficiently synthesizing chemicals from previously unadapted renewable substrates. By carefully designing gene knockout strategies that couple cell growth and substrate assimilation, we can increase the evolutionary driving force. This strategy has been successfully demonstrated in creating methylotrophic *E. coli* strains that efficiently utilize methanol as the sole carbon source [9] and in *C. glutamicum* to achieve coutilization of methanol and xylose [14].

Computational tools can significantly enhance the design of starting strains for the ALE process [15]. However, current methods are limited by their reliance on small-scale core metabolic models to avoid combinatorial explosion, making exhaustive searches impractical in genome-scale models [5]. For example, Keller et al. used the *E. coli* core model to evaluate 33 gene knockout combinations across 11 cosubstrates, resulting in over 2 million in silico experiments using flux balance analysis (FBA) [16,17]. However, the comprehensive genome-scale metabolic model of *E. coli*, iML1515, with 2,712 reactions, makes exhaustive searches impractical. Furthermore, no computational work has assessed the minimal requirement of unadapted carbon sources for the initial strain or the substrate assimilation driving force (SADF) under the designed genotypes. Many existing strain design algorithms—such as OptKnock [18], OptForce [19], OptCouple [20], QHEPath [21], and Minimal Cut Sets [22]—focus primarily on coupling microbial growth to overproduction of a target product. These methods are optimized for producing high titers of specific products. In contrast, the method we are introducing, called “AdaptUC”, addresses a fundamentally different objective: facilitating growth on previously unadapted carbon sources, like methanol or formate. Instead of maximizing product flux, AdaptUC maximizes the SADF and enforces metabolic dependency on the novel substrate. By doing so, it provides a new computational tool that accelerates ALE toward novel carbon utilization—a goal not supported by traditional strain design frameworks focused on product synthesis.

AdaptUC comprises 2 variants of bilevel mixed-integer programming (MIP) algorithms. AdaptUC is designed to uncover insights into microbial carbon source adaptation by identifying gene knockout strategies that serve as an evolutionary driving force for strains to utilize previously unadapted carbon sources. By transforming all considerations for the design of the initial strain into internal constraints that must be simultaneously satisfied, AdaptUC can identify knockout targets that couple the target substrate with growth in genome-scale metabolic models containing thousands of reactions. Two quantitative metrics are used to evaluate the predicted strategies: the minimal requirement of the unadapted carbon source for the starting strain and the carbon assimilation driving force correlated with the degree of growth coupling.

We demonstrate the effectiveness of AdaptUC through case studies involving important industrial species, *E. coli* and *C. glutamicum*, focusing on methanol utilization both in coutilization with other carbon sources and as the sole carbon source. We validate our method by comparing the prediction results with experimentally effective strategies reported in the literature. Furthermore, we present new strategies with superior potential, characterized by higher evolutionary driving forces and lower initial substrate requirements, found through an in-depth metabolic-level analysis. Our findings reveal that the proportion of biomass precursors that must be synthesized from unadapted carbon sources is a critical determinant of the evolutionary driving force. By leveraging computational analysis through AdaptUC, we can predict metabolic engineering strategies for ALE, enabling microorganisms to coutilize an adapted cosubstrate and an unadapted carbon source or, in some cases, exclusively rely on the unadapted source.

## Results and Discussion

### Design of AdaptUC

**Fig. 1.**
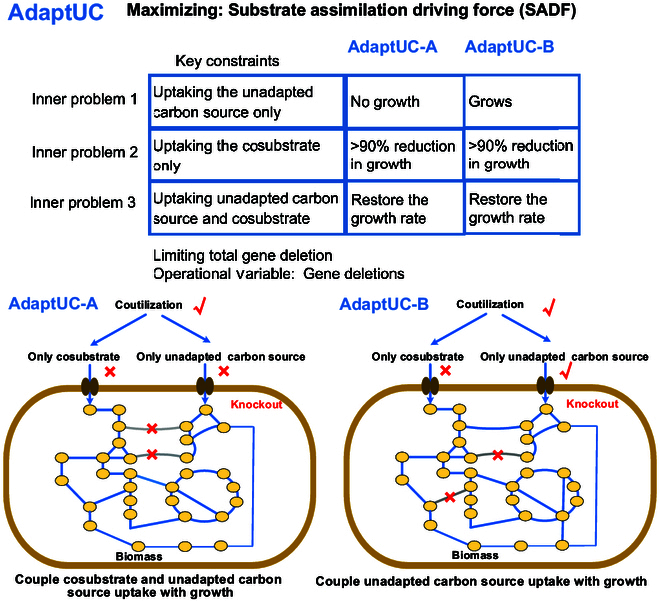
The schematic diagram for the concept of AdaptUC.

In this study, we present a computational framework named AdaptUC, designed to predict gene knockout strategies for developing a starting strain for the ALE process. The goal is to enable the utilization of an unadapted carbon source. As depicted in Fig. 1, AdaptUC includes 2 algorithms: AdaptUC-A and AdaptUC-B. AdaptUC-A, with its concept initially introduced by Gawand et al. [23], couples strain growth with both the unadapted substrate and a cosubstrate, requiring simultaneous supply of both for growth, and while AdaptUC-A shares the idea of Gawand et al.’ s SIMUP algorithm, we optimize the SADF rather than biomass flux. SADF instead captures the evolutionary driving force underlying ALE. To our knowledge, SADF is a novel objective that better aligns with experimental ALE protocols and provides a rational design principle for engineering strains to utilize previously unadapted carbon sources. AdaptUC-B, a new bilevel algorithm, couples strain growth exclusively with the unadapted substrate, enabling growth only when the unadapted substrate is present. AdaptUC-A predicts knockout targets that result in the strain requiring both the unadapted substrate and the adapted cosubstrate for growth. This approach allows the strain to gradually adapt to the unadapted substrate during the evolutionary process, although it may still be unable to grow on the previously unadapted substrate as the sole carbon source. In contrast, AdaptUC-B preserves the potential for utilizing the unadapted substrate as the sole carbon source. Both AdaptUC-A and AdaptUC-B include 3 inner problems (design requirements), each with an objective that must satisfy the constraints in the outer level with minimal number of modifications (Fig. 1). Each inner problem and its corresponding outer-level constraint represent a requirement for the ALE starting strain, delineating the expectations for the strain at the conclusion of the ALE process. As shown in the table in Fig. 1, these 3 inner problems are as follows:

1. Growth on the unadapted substrate alone:

AdaptUC-A: no growth when only the unadapted carbon source is available.

AdaptUC-B: robust growth when only the unadapted carbon source is available

2. Dependence on the unadapted substrate:

Upon provision of only the cosubstrate, growth must drop by at least 90% relative to the reference strain’s maximum growth on the unadapted substrate (prior to any gene deletions).

3. Restoration of growth via cosubstrate supplementation:

When the unadapted substrate is added to a medium containing the cosubstrate, the strain’s growth rate must recover to its predeletion level.

**Fig. 2.**
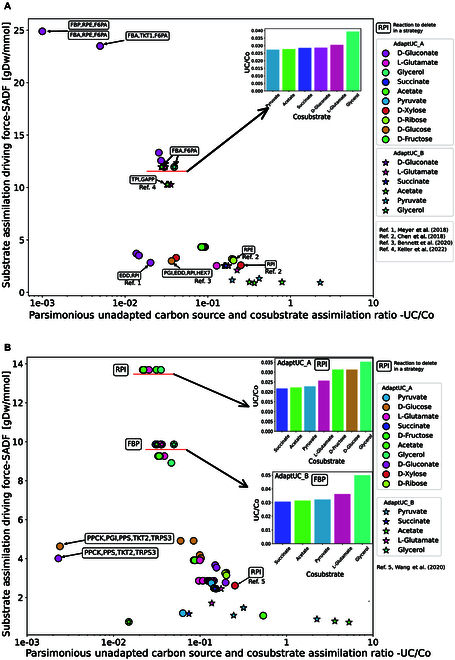
Substrate assimilation driving force of AdaptUC predicted strategies for designing methylotrophic (A) *E. coli* and (B) *C. glutamicum.* Methanol is the unadapted carbon source and the cosubstrates are listed in the legend. Successful strategies reported by literature are annotated. Original data presented in Tables S14 and S15.

The first inner problem signifies the necessity for the strain after ALE to exhibit no growth (AdaptUC-A) or robust growth (AdaptUC-B) on a medium with an unadapted substrate as the sole carbon source (Fig. 2). The second requirement dictates that the strain after ALE should not thrive well without the unadapted carbon source, even in the presence of the cosubstrate. Combining these 2 requirements effectively couples the assimilation of the unadapted carbon source with growth, generating a compelling driving force for the cell to uptake the unadapted substrate. In the final inner problem, where the unadapted substrate is available for uptake, as opposed to the second inner problem, the objective is to ensure that the optimal growth of the strain after ALE is not inferior to the growth of the reference strain on the cosubstrate. This design requirement guarantees that the addition of cosubstrate to the medium containing the unadapted carbon source can aid the survival and growth of the starting strain in the ALE process. We assume that the gene deletions for the strain after ALE and the ALE starting strain are either similar or that their differences do not significantly impact growth. AdaptUC is specifically designed to construct the ALE starting strain. The algorithm details are provided in Methods.

### Designing metabolic engineering strategies for creating methylotrophic strains and literature validation

We applied AdaptUC to predict gene modifications for developing methylotrophic *E. coli* strains. Methanol serves as an unadapted carbon source for *E. coli*, which naturally lacks a methanol assimilation pathway. The mere insertion of a heterologous methanol assimilation pathway does not immediately grant *E. coli* the ability to grow on a medium where methanol is the sole carbon source [9]. This necessitates gene modifications and ALE experiments to induce *E. coli* to evolve the capacity for methanol assimilation. To facilitate this, we first introduced the methanol assimilation pathway (comprising methanol dehydrogenase, 3-hexulose 6-phosphate synthase, and 6-phospho 3-hexulose isomerase) into the most recent *E. coli* K12 model, *iML1515*. We then used AdaptUC to predict gene modification strategies for constructing the initial strain. To ensure the initial strain’s survival in the initial ALE media, we considered 10 cosubstrates (succinate, fructose, glucose, acetate, glutamate, glycerol, glycerate, pyruvate, xylulose, and ribose). The number of allowable knockout reactions was limited to no more than 5. Our 89 candidate designs were scored by 2 metrics:

UC/Co ratio (*x* axis in Fig. 2A)—the minimal uptake flux of methanol relative to the cosubstrate required to sustain growth, reflecting how parsimoniously the unadapted carbon source is used (see details in Methods).

SADF (*y* axis in Fig. 2A)—the average SADF, defined as the incremental growth rate gained per unit increase in methanol uptake over a spectrum of fluxes, quantifying the evolutionary pressure toward methanol assimilation (see details in Methods).

AdaptUC successfully predicted 5 experimentally validated strategies for methylotrophic *E. coli* strains (Table S1). Four of these are AdaptUC-A strategies, disrupting the RuMP cycle to prevent the formaldehyde acceptor ru5p from being generated by methanol alone, while preventing the cosubstrate from entering the Embden–Meyerhof–Parnas (EMP) or Entner–Doudoroff (ED) pathway without methanol, forcing the cosubstrate to be the sole precursor of ru5p. The validated RuMP-interrupted strategies include xylose-cosubstrate-Δ*rpi* [24], ribose-cosubstrate-Δ*rpe* [24], glucose-cosubstrate-Δ*pgi*Δ*edd*Δ*rpi* [25], and gluconate-cosubstrate-Δ*edd*Δ*rpi* [26]. An AdaptUC-B strategy, using pyruvate as the cosubstrate and deleting *tpi*, was also validated by literature [9]. Due to the limitation of the stoichiometric model, there are minor differences between literature and the predicted genotype, which are explained in the Supplementary Materials.

We predicted strategies with much higher SADF and lower UC/Co than reported strategies. For *E. coli*, deletion of *fba*, *rpe*, and *f6pa* while using gluconate as the cosubstrate shows a more than 5-fold higher driving force than currently reported AdaptUC-A strategies (Fig. 2A) with the same deletions. The selection of cosubstrate can significantly affect SADF. For the AdaptUC-B strategies, we predict that the pyruvate-Δ*fba,* Δ*f6pa* strategy has slightly higher SADF and lower UC/Co than the experimentally successful cosubstrate-pyruvate-ΔTPI strategy.

For *C. glutamicum*, there are significantly fewer reported cases compared to *E. coli*. Deletion of ribose-5-phosphate isomerase (*rpi*) and using xylose as a substrate can achieve coutilization of xylose and methanol, a reported case that has been predicted by AdaptUC. However, similar to the situation in *E. coli*, we found that in the deactivation of *rpi*, xylose can only be converted to ru5p and combine with formaldehyde in a 1:1 ratio, causing the uptake of formaldehyde and xylose to be completely coupled. Compared to strategies that decouple methanol from the cosubstrate, achieving the same growth rate with this strategy requires more methanol, resulting in a lower SADF. We also predicted that simply changing the cosubstrate while keeping the *rpi* deletion can significantly decouple methanol from the cosubstrate, improving SADF and lowering UC/Co. Predictions show that using 7 different substrates, such as succinate, acetate, and pyruvate, can achieve this decoupling effect, increasing SADF by 5.2 times and reducing UC/Co by 91% (Fig. 2B).

*C. glutamicum* requires fewer reaction deletions compared to *E. coli*. This is because *C. glutamicum* lacks the ED pathway and some gluconeogenesis reactions present in *E. coli.* For example, within the AdaptUC-B strategy, the preferred approach is the deletion of *FBP*. This strategy allows for the production of fructose-6-phosphate, which is necessary for cell wall synthesis, without disrupting the exclusive utilization of methanol to produce it. Additionally, there is no need to delete an alternative gluconeogenesis fructose 6-phosphate aldolase (*F6PA*) because it is not present in *C. glutamicum*.

**Fig. 3.**
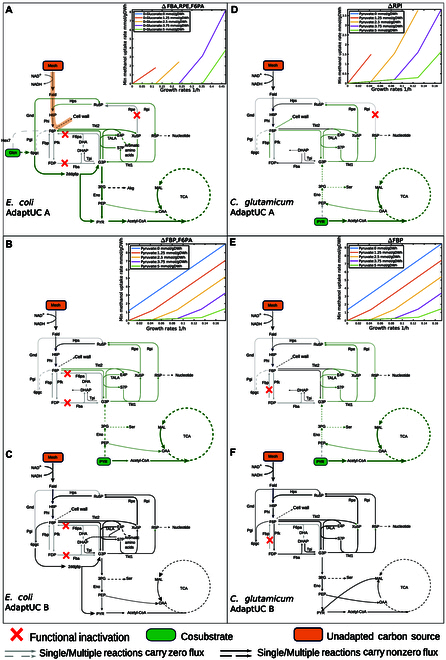
Selected promising strategies for interrupted (A and D) and uninterrupted (B, C, E, and F) RuMP cycle corresponding to AdaptUC-A and AdaptUC-B strategies, respectively. For AdaptUC-B strategies, panels (B) and (E) show the flux diagrams before evolution, where the cosubstrate and unadapted carbon source are utilized simultaneously. Panels (C) and (F) show the flux diagrams after evolution, where methanol (the unadapted carbon source) is used as the sole carbon source. The smaller plots in the top right of each subfigure depict the minimal uptake rates of methanol as they change with growth rate under different cosubstrate uptake rates.

Simplified metabolic flux diagrams have been prepared for 4 unreported promising strategies for *E. coli* and *C. glutamicum* (Fig. 3). The top right of each subfigure shows the minimal uptake rates of methanol as a function of growth rate under different cosubstrate uptake rates. A positive reciprocal of the slope indicates the degree of growth coupling with methanol uptake rates. The slope may change with increasing growth rates, signifying a switch in metabolic flux patterns. As the slope increases, it indicates that cosubstrates are becoming insufficient, and the unadapted carbon source must be utilized to synthesize more biomass precursors. During the transition from growing on both cosubstrates and the unadapted substrate to growing solely on the previously unadapted substrate, the flux direction in the lower EMP pathway may change, requiring regulatory adjustments during the ALE process (Fig. 3B and C, and E and F).

### A workflow combining AdaptUC-A and AdaptUC-B

**Fig. 4.**
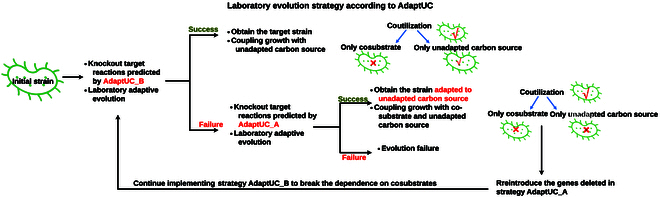
The proposed workflow combines AdaptUC-A and AdaptUC-B to maximize the success of producing strains capable of growing on a medium with an unadapted substrate as the sole carbon source.

We observed that for both *E. coli* and *C. glutamicum*, AdaptUC-A strategies can yield higher SADF and lower UC/Co ratios compared to AdaptUC-B strategies (Fig. 2). There are overlaps in targets within AdaptUC-A and AdaptUC-B strategies. For example, one of the best AdaptUC-A strategies for *E. coli* with gluconate as a cosubstrate involves Δ*fba*Δ*rpe*Δ*f6pa*, achieving an SADF of 24.9 and a UC/Co of 0.001. In contrast, one of the best AdaptUC-B strategies for *E. coli* using pyruvate as a cosubstrate involves Δ*fba*Δ*f6pa*, with a lower SADF of 11.9 and a higher UC/Co of 0.027. This indicates that if AdaptUC-B strategies fail, we can switch to an AdaptUC-A strategy that requires less unadapted carbon source (lower UC/Co) and has a higher evolutionary driving force (SADF). Through this process, the strain can adapt to the unadapted substrate more easily. If the AdaptUC-A strategy is successful in achieving a strain that grows solely on an unadapted carbon source, minimal adjustments can be made by comparing both AdaptUC-A and AdaptUC-B strategies. In this example, *rpe* should be reintroduced to the strain, and the cosubstrate should be gradually changed to pyruvate, followed by evolving the Δ*fba*Δ*f6pa* strain, improving the strain’s growth dependence on the unadapted substrate while the cosubstrate being gradually reduced to zero. We have summarized the workflow in Fig. 4 to maximize the success of producing strains capable of growing on a medium with an unadapted substrate as the sole carbon source.

### The degree of growth coupling of unadapted carbon source determines the evaluation metrics of strategies

**Fig. 5.**
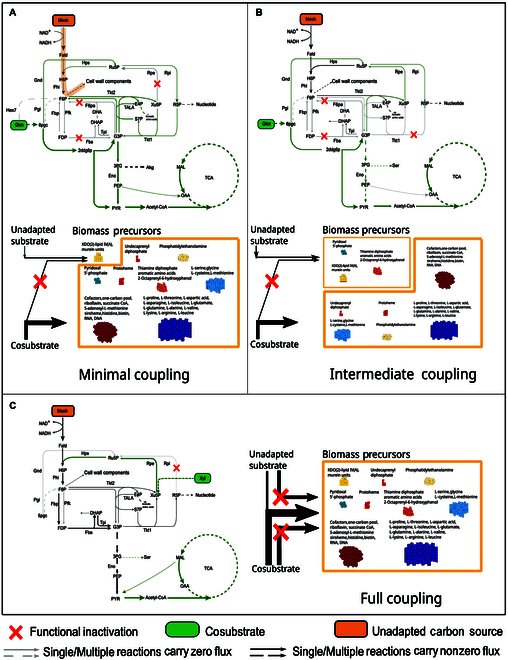
Mode of unadapted carbon source coupling to biomass. (A) Minimal coupling, only a minimal portion of biomass must be synthesized with an unadapted carbon source. (B) Intermediate coupling, multiple but not all biomass components must be synthesized with an unadapted carbon source. (C) Full coupling, no biomass components can be produced without the unadapted carbon source.

A desirable strategy is characterized by a high SADF and a low UC/Co ratio. From Fig. 2, it can be observed that the strategies are not evenly distributed in the SADF and UC/Co panel but are clustered together. The SADF and UC/Co values of strategies within different clusters vary significantly. By analyzing metabolic flux pathways, we discovered that the substantial differences in SADF and UC/Co values among strategies in different clusters are due to the varying degrees of coupling between the unadapted substrate and cell growth. Strategies clustered together in Fig. 2 exhibit similar degrees of growth coupling, meaning that the same or similar biomass precursors must be synthesized using the unadapted substrate. Both AdaptUC-A and AdaptUC-B ensure that not all biomass components can be synthesized solely from the cosubstrate to create a certain level of substrate utilization driving force. Consequently, the growth coupling degree of the unadapted substrate in all predicted strategies ranges from 0% to 100%. A low degree of growth coupling indicates that only a small portion of the biomass requires the unadapted substrate for synthesis. In such cases, the UC/Co ratio is low, suggesting that the initial strain can easily survive on media containing the unadapted substrate. Simultaneously, most of the biomass is synthesized from the cosubstrate, which the cell can assimilate in large amounts. This means that even a small uptake of the unadapted substrate can significantly boost biomass production, resulting in a high SADF. We classify strategies into 3 categories based on the degree of growth coupling with the unadapted substrate: minimal coupling, intermediate coupling, and full coupling. Full coupling means that the synthesis of all biomass precursors depends entirely on the unadapted substrate (Fig. 5C).

**Fig. 6.**
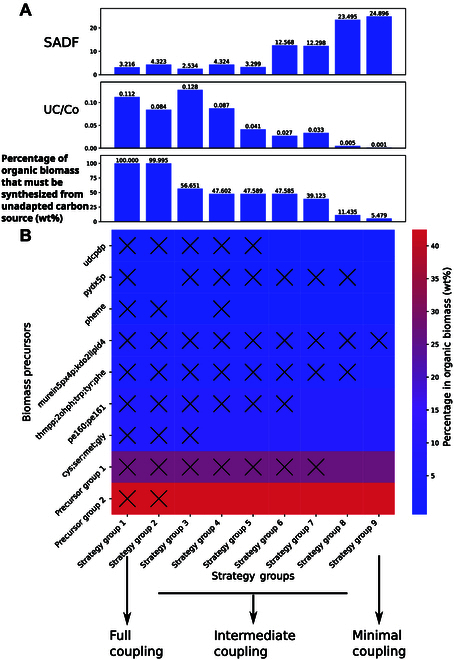
Biomass precursors producibility from cosubstrate for AdaptUC-A predicted strategies. (A) The SADF, UC/Co, and percentage of biomass precursors that must be synthesized with unadapted precursors. (B) The color in the heat map indicates the mass percentage of precursor groups in total organic cellular biomass of *E. coli*, and the cross mark indicates that the corresponding biomass precursor group cannot be synthesized by cosubstrate alone (or must be synthesized by the unadapted carbon source) under the corresponding strategy group in the *x* axis. Precursor group 1: biotin, riboflavin, succoa, amet, thf, mlthf, 10fthf, fad, sheme, nadp, coa, nad, dttp, dctp, datp, dgtp, histidine, ctp, utp, and gtp. Precursor group 2: pro, thr, asp, asn, ile, glu, gln, ala, val, lys, arg, and leu. Strategy group 9 (minimal coupling): glcn: FBA, RPE, and F6PA; glcn: FBP, RPE, and F6PA. Strategy group 1 (full coupling): xyl: TKT1; xyl: RPI, etc.; 16 strategies altogether. The full content of each strategy group can be found in Tables S6 and S7.

Specifically, the minimal coupling case for *E. coli* involves using gluconate as a substrate and knocking out *fba* (fructose-bisphosphate aldolase), *f6pa* (fructose 6-phosphate aldolase), and *rpe* (ribulose 5-phosphate 3-epimerase) (Fig. 5A). In this scenario, due to the deletion of *fba* and *f6pa*, gluconate-derived glyceraldehyde 3-phosphate cannot produce D-fructose 6-phosphate. However, D-fructose 6-phosphate is an irreplaceable precursor for KDO(2)-lipid IV (A) and murein units, which contribute respectively to the peptidoglycan layer and lipopolysaccharide structure of the cell wall. KDO(2)-lipid IV(A) and murein units represent only 5.48 wt% of the biomass. Therefore, D-fructose 6-phosphate synthesis must occur through formaldehyde (derived from the unadapted substrate methanol) reacting in a 1:1 molar ratio with D-ribulose 5-phosphate. The latter is entirely derived from gluconate, but only accounts for 0.63% of the gluconate pathway. The remaining 92.3% of the carbon flux channels through the ED pathway toward glyceraldehyde 3-phosphate and downstream glycolysis and the tricarboxylic acid (TCA) cycle, while 7.1% converts to D-ribose 5-phosphate, which later channels into nucleic acid synthesis. The synthesis of KDO(2)-lipid IV(A) and murein units, which make up only 5.48 wt% of the biomass, requires methanol (Fig. 6A and B). Due to the low proportion of these biomass precursors and the fact that 83% of the carbon comes from gluconate, this minimal coupling strategy results in a very low UC/Co (0.001) and a high SADF (24.9 gDW/mmol).

In contrast, a full coupling scenario has a much lower driving force and a much higher UC/Co ratio. For example, in the case of deleting *RPI* while using D-xylose as the cosubstrate (Fig. 5C), D-xylose cannot synthesize biomass precursors independently but channels to D-ribulose 5-phosphate, which can only combine with formaldehyde to form hexulose 6-phosphate in a 1:1 molar ratio. This results in no biomass precursor being synthesized without methanol (Fig. 6A and B, the leftmost column). Most predicted strategies result in intermediate coupling to growth (Figs. 5B and 6A and B, middle columns). Generally, SADF increases and UC/Co decreases with the decrease of the degree of the unadapted carbon coupling with growth. Strategies with low growth coupling are advantageous (Fig. 6A).

## Conclusion

AdaptUC, including AdaptUC-A and AdaptUC-B, share much of the same MILP backbone, but they pursue distinct engineering goals. AdaptUC-A requires both substrates for growth (driving coutilization), whereas AdaptUC-B’s added constraint breaks that dependency, achieving exclusive growth on the novel carbon sources. AdaptUC facilitates the design of starting strains for ALE by predicting gene knockout strategies that serve as evolutionary driving forces for utilizing previously unadapted carbon sources. Specifically, in the context of methanol utilization, AdaptUC not only predicts experimentally validated strategies but also identifies novel strategies with enhanced evolutionary driving forces and reduced initial methanol requirements for both coutilization and sole utilization scenarios.

Our findings underscore the critical role of the proportion of biomass precursors synthesized from unadapted carbon sources in determining both the evolutionary driving force and minimal substrate requirements. Through AdaptUC, we leverage computational analysis to predict metabolic engineering strategies for ALE, enabling microorganisms to either coutilize an adapted cosubstrate with an unadapted carbon source or, in some instances, exclusively rely on the unadapted source.

This method holds the potential to revolutionize industrial biosynthesis by facilitating more efficient utilization of renewable carbon sources. Moreover, establishing a strain library through the integration of high-throughput automated biofoundry systems with strain construction scheduling optimization algorithms ([27,28] and demonstrated in Ref. [29]) offers promising avenues for enhancing the quality of metabolic models. For example, integrating the hierarchical dual-core multitask learning framework described in Ref. [29] for predicting enzyme commission numbers could enhance AdaptUC’s ability to accurately predict enzyme functions—a key component in devising effective metabolic engineering strategies. Additionally, incorporating thermodynamic and enzyme-constrained methods into metabolic models can improve phenotype predictions by accounting for the energetic feasibility and kinetic constraints of enzymatic reactions [30,31], thus providing a more realistic representation of cellular metabolism. Such enhancements to the foundational metabolic models would further improve AdaptUC’s performance, leading to more effective metabolic engineering strategies.

Looking ahead, the adaptation of microbial strains to unadapted carbon sources via AdaptUC opens exciting avenues for sustainable bioproduction. Once adapted, these strains can be further engineered to produce valuable chemicals. To optimize production yields, advanced metabolic engineering tools can be employed to design metabolic networks. For instance, growth-coupling strategies like OptKnock [18,20,32,33] can link the production of target compounds to cell growth, ensuring high production rates. Moreover, as highlighted in Refs. [34,35], designing production pathways that are bioorthogonal to native cellular reactions can minimize interference and enhance bioproduction efficiency. This approach not only improves yields but also fosters the development of novel biocatalytic systems, paving the way for a more sustainable bioeconomy.

## Methods

### Development of AdaptUC

The 2 components of AdaptUC framework, AdaptUC-A and AdaptUC-B, each encompasses 3 requirements that must be satisfied simultaneously. The first design requirement is that the strain after gene modification has the potential to evolve to a phenotype that can grow on the untapped carbon source. The growth rate is simulated by FBA.

Maximize $v1^{\mathrm{bio}}=\mathrm{cv}1$

Subject to Eqs. 1 to 5

$$\sum_{j\in J}S_{i,j}\;v1_{j}=0,\;\forall i\in I$$ (1)

$$\begin{array}{cc} \mathrm{LB}1_{j}\leq v1_{j}\leq \mathrm{UB}1_{j} & \forall j\in J \end{array}$$ (2)

$$\begin{array}{cc} \mathrm{LB}1_{j}\left(1-y_{j}\right)\leq v1_{j}\leq \mathrm{UB}1_{j}\left(1-y_{j}\right) & \forall j\in J^{\mathrm{cd}} \end{array}$$ (3)

$$\begin{array}{cc} \mathrm{LB}1_{j}=-10 & \forall j\in J^{\mathrm{uc}} \end{array}$$ (4)

$$\begin{array}{cc} \mathrm{LB}1_{j}=0 & \forall j\in J^{\text{cosub}} \end{array}$$ (5)

where $S_{i,j}$ denotes the stoichiometric coefficient matrix of metabolite *i* in reaction *j*, $v1_{j}$ is the flux variable of reaction *j* for the first design requirement, and $\mathrm{LB}1_{j}$ and $\mathrm{UB}1_{j}$ are the lower and upper bounds for $v1_{j}$**;** these bounds can be used to setup reactions’ reversibility based on thermodynamics. The binary variable $y_{j}$ indicates whether the reaction *j* is deactivated ($y_{j}=0$) or not ($y_{j}=0$).

The outer level constraint on the first inner problem is that, for the AdaptUC B variant, it must still can grow on the unadapted substrate after deletion, and the reduction of growth due to gene deletion must not exceed 50%:

$$v1^{\mathrm{bio}}\geq 0.5v0^{\mathrm{bio}\_\mathrm{uc}}$$ (6)

where $v0^{\mathrm{bio}\_\mathrm{uc}}$ is the potential maximal growth rate of reference strain on the untapped carbon source that can be calculated by solving the following FBA problem:

Maximize $v0^{\mathrm{bio}\_\mathrm{uc}}=c^{T}v$

Subject to Eqs. 7 to 9

$$\begin{array}{cc} \sum_{j\in J}S_{i,j}\;v_{j}=0, & \forall i\in I \end{array}$$ (7)

$$\begin{array}{cc} \mathrm{LB}1_{j}\leq v_{j}\leq \mathrm{UB}1_{j} & \forall j\in J \end{array}$$ (8)

$$\begin{array}{cc} \mathrm{LB}1_{j}=-10 & \forall j\in J^{\mathrm{uc}} \end{array}$$ (9)

where $J^{\mathrm{uc}}$ are uptake reactions for the unadapted substrate and $c^{T}$ is transpose of the objective coefficient vector, which has a length equal to the number of model reactions, with all entries set to zero except a value of 1 at the index corresponding to the biomass reaction. Thus, the objective function $c^{T}$ directly maximizes the flux through the biomass reaction, which is equivalent to maximizing the simulated growth rate using FBA

For the AdaptUC A variant, the deletion causes the strain being not able to grow on the unadapted substrate. We set the growth reduction greater than 90% to achieve this constraint.

$$v1^{\mathrm{bio}}\leq 0.1v0^{\mathrm{bio}\_\mathrm{uc}}\;$$ (10)

The second requirement is that after deletion, the strain suffers no growth or severely impaired growth on conventional cosubstrate, forcing the strain to utilize C1-substrate whenever it is available. Considering 90% reduction in growth rate fulfills this requirement, then this requirement is implemented in the following constraint:

$$v2^{\mathrm{bio}}\leq 0.1v0^{\mathrm{bio}\_\text{cosub}}$$ (11)

The calculation for growth rate of the reference strain on cosubstrate ($v0^{\mathrm{bio}\_\text{cosub}}\;$) is similar to $v0^{\mathrm{bio}\_\mathrm{uc}}$, and the difference is to set the bound for cosubstrate exchange reaction instead. The growth rate $v2^{\mathrm{bio}}$ is calculated by the following inner problem:

Maximize $v2^{\mathrm{bio}}=c^{T}v2$

Subject to Eqs. 12 to 16

$$\begin{array}{cc} \sum_{j\in J}S_{i,j}\;v2_{j}=0, & \forall i\in I \end{array}$$ (12)

$$\begin{array}{cc} \mathrm{LB}2_{j}\leq v2_{j}\leq \mathrm{UB}2_{j} & \forall j\in J \end{array}$$ (13)

$$\begin{array}{cc} \mathrm{LB}2_{j}\left(1-y_{j}\right)\leq v2_{j}\leq \mathrm{UB}2_{j}\left(1-y_{j}\right) & \forall j\in J^{\mathrm{cd}} \end{array}$$ (14)

$$\begin{array}{cc} \mathrm{LB}2_{j}=0 & \forall j\in J^{C1} \end{array}$$ (15)

$$\begin{array}{cc} \mathrm{LB}2_{j}=-10 & \forall j\in J^{\text{cosub}} \end{array}$$ (16)

The third requirement is that addition of unadapted substrate to the cosubstrate-only medium can recover the growth rate to the growth before gene deletion. This requirement is implemented by the following outer-level constraint:

$$v3^{\mathrm{bio}}\geq v0^{\mathrm{bio}\_\text{cosub}}$$ (17)

where the growth rate $v3^{\mathrm{bio}}$ is calculated by the third inner problem:

Maximize $v3^{\mathrm{bio}}=c^{T}v3$

Subject to Eqs. 18 to 22

$$\begin{array}{cc} \sum_{j\in J}S_{i,j}\;v3_{j}=0, & \forall i\in I \end{array}$$ (18)

$$\begin{array}{cc} \mathrm{LB}3_{j}\leq v3_{j}\leq \mathrm{UB}3_{j} & \forall j\in J \end{array}$$ (19)

$$\begin{array}{cc} \mathrm{LB}3_{j}\left(1-y_{j}\right)\leq v3_{j}\leq \mathrm{UB}3_{j}\left(1-y_{j}\right) & \forall j\in J^{\mathrm{cd}} \end{array}$$ (20)

$$\begin{array}{cc} \mathrm{LB}3_{j}=-10 & \forall j\in J^{\mathrm{uc}} \end{array}$$ (21)

$$\begin{array}{cc} \mathrm{LB}3_{j}=-10 & \forall j\in J^{\text{cosub}} \end{array}$$ (22)

The minimal uptake rate for the unadapted carbon source to recover growth after gene deletion, *v*^UC^ can be obtained by the following inner problem:

Minimize $v^{c1}=-g^{T}v4$

Subject to

$$\begin{array}{c} \left[\begin{array}{c} \;\sum_{j\in j}S_{i,j}\;v4_{j}=0\;\forall i\in I\\ \mathrm{LB}4_{j}\leq v4_{j}\leq \mathrm{UB}4_{j}\;\forall j\in J\\ \mathrm{LB}4_{j}\left(1-y_{j}\right)\leq v4_{j}\leq \mathrm{UB}4_{j}\left(1-y_{j}\right)\;\forall j\in J^{\mathrm{cd}}\\ \mathrm{LB}4_{j}=-1000\;\forall j\in J^{\mathrm{uc}}\\ \mathrm{LB}4_{j}=-10\;\forall j\in J^{\text{cosub}}\\ \mathrm{cv}4\geq v0^{\mathrm{bio}\_\text{cosub}} \end{array}\right] \end{array}$$ (23)

where $g^{T}$ is transpose of the objective coefficient vector, which has a length equal to the number of model reactions, with all entries set to zero except a value of 1 at the index corresponding to the unadapted substrate exchange reaction.

The total number of reaction deletion must be constrained to accelerate the solving process:

$$\sum_{j\in J^{\mathrm{cd}}}y_{j}<K$$ (24)

This constraint (Eq. 24) helps balance computational tractability and experimental feasibility, as excessive knockouts may compromise cell viability or lead to unintended phenotypes. The upper bound *K* can be adjusted by users based on the desired design complexity and organism-specific considerations.

Maximize $\bar{\text{SADF}}$

Maximize$v1^{\mathrm{bio}}=c^{T}v1$

Subject to Eqs. 25 to 32

$$\begin{array}{c} \left[\begin{array}{c} \sum_{j\in J}S_{i,j}\;v1_{j}=0,\;\forall i\in I\\ \mathrm{LB}1_{j}\leq v1_{j}\leq \mathrm{UB}1_{j}\;\forall j\in J\\ \mathrm{LB}1_{j}\left(1-y_{j}\right)\leq v1_{j}\leq \mathrm{UB}1_{j}\left(1-y_{j}\right)\;\forall j\in J^{\mathrm{cd}}\\ \mathrm{LB}1_{j}=-10\;\forall j\in J^{\mathrm{uc}}\\ \mathrm{LB}1_{j}=0\;\forall j\in J^{\text{cosub}} \end{array}\right] \end{array}$$ (25)

Maximize $v2^{\mathrm{bio}}=c^{T}v2$

Subject to

$$\begin{array}{c} \left[\begin{array}{c} \sum_{j\in J}S_{i,j}\;v2_{j}=0,\;\forall i\in I\\ \mathrm{LB}2_{j}\leq v2_{j}\leq \mathrm{UB}2_{j}\;\forall j\in J\\ \mathrm{LB}2_{j}\left(1-y_{j}\right)\leq v2_{j}\leq \mathrm{UB}2_{j}\left(1-y_{j}\right)\;\forall j\in J^{\mathrm{cd}}\\ \mathrm{LB}2_{j}=0\;\forall j\in J^{\mathrm{uc}}\\ \mathrm{LB}2_{j}=-10\;\forall j\in J^{\text{cosub}} \end{array}\right] \end{array}$$ (26)

Maximize $v3^{\mathrm{bio}}=\mathrm{cv}3$

Subject to

$$\begin{array}{c} \left[\begin{array}{c} \sum_{j\in J}S_{i,j}\;v3_{j}=0,\;\forall i\in I\\ \mathrm{LB}3_{j}\leq v3_{j}\leq \mathrm{UB}3_{j}\;\forall j\in J\\ \mathrm{LB}3_{j}\left(1-y_{j}\right)\leq v3_{j}\leq \mathrm{UB}3_{j}\left(1-y_{j}\right)\;\forall j\in J^{\mathrm{cd}}\\ \mathrm{LB}3_{j}=-10\;\forall j\in J^{\mathrm{uc}}\\ \mathrm{LB}3_{j}=-10\;\forall j\in J^{\text{cosub}} \end{array}\right] \end{array}$$ (27)

Minimize $v^{c1}=-g^{T}v4$

Subject to

$$\begin{array}{c} \left[\begin{array}{c} \sum_{j\in J}S_{i,j}\;v4_{j}=0,\;\forall i\in I\\ \mathrm{LB}4_{j}\leq v4_{j}\leq \mathrm{UB}4_{j}\;\forall j\in J\\ \mathrm{LB}4_{j}\left(1-y_{j}\right)\leq v4_{j}\leq \mathrm{UB}4_{j}\left(1-y_{j}\right)\;\forall j\in J^{\mathrm{cd}}\\ \mathrm{LB}4_{j}=-1,000\;\forall j\in J^{\mathrm{uc}}\\ \mathrm{LB}4_{j}=-10\;\forall j\in J^{\mathrm{cosub}}\\ \mathrm{cv}4\geq v0^{\mathrm{bio}\_\text{cosub}} \end{array}\right] \end{array}$$ (28)

$$v1^{\mathrm{bio}}\leq 0.1\;v0^{\mathrm{bio}\_\mathrm{uc}}\;\mathrm{for}\;\mathrm{AdaptUC}\;\text{B}$$ (29)

$$v1^{\mathrm{bio}}\leq 0.1\;v0^{\mathrm{bio}\_\mathrm{uc}}\;\mathrm{for}\;\mathrm{AdaptUC}\;\text{A}$$ (30)

$$v2^{\mathrm{bio}}\leq 0.1\;v0^{\mathrm{bio}\_\text{cosub}}$$ (31)

$$v3^{\mathrm{bio}}\geq v0^{\mathrm{bio}\_\text{cosub}}$$ (32)

$$v1.v2,v2,v4\in R,y\in \left[0,1\right]$$ (33)

In summary, the algorithm AdaptUC is then formulated as below:

The MILP was formulated and solved using MATLAB (R2019b) with the COBRA Toolbox (version 2.42.0) and the IBM CPLEX solver (version 12.10). All case studies were performed on a machine running CentOS 7 with an Intel Xeon 8375C CPU. To convert the bilevel optimization problem into a single-level MILP, we employed the Karush–Kuhn–Tucker conditions, including the strong duality constraint, which ensures that the optimal objective values of the primal and dual problems are equal (inner problem 1 as an example shown in Tables S8 to S10). The single-level version of AdaptUC for optimization is shown in Table S11. Complementary slackness simplification and Charnes–Cooper transformation are used to ensure linearity of the entire problem (as shown in Tables S12 and S13). To identify multiple solutions, including suboptimal but valid alternatives, we iteratively applied integer cuts to exclude previously identified solutions and explore new equivalent or near-optimal ones, until no more solutions are found. The integer cut constraint is:

$$\sum_{j\in J^{\text{positive}-\text{last}-\text{round}}}y_{j}<N-1$$ (34)

where $J^{\text{positive}-\text{last}-\text{round}}$ is the set of the index of knockouts in last round of AdaptUC solution, and *N* is the length of $J^{\text{positive}-\text{last}-\text{round}}$.

To ensure AdaptUC remains tractable on full genome-scale reconstructions, we apply 2 key strategies: (a) exploiting the branch-and-bound engine of modern MIP solvers to prune infeasible regions of the search space, and (b) prefiltering the full reaction set by removing zero-flux reactions, gene-unassociated reactions, peripheral secondary-metabolism pathways, etc., as introduced by Balagurunathan et al. [36]. For example, this can yield 92 candidate knockouts for *E. coli* with xylose as cosubstrate and methanol as the unadapted substrate, as shown in Table S16.

To quantify these improvements, we benchmarked AdaptUC-B on an Intel Xeon 8375C CPU and CPLEX solver 12.10. For each knockout limit *N* = 2, 3, …, 8, we generated 40 optimal solutions and measured runtimes, as shown in Fig. S2. Even for *N* = 8, the average runtime remains under 1 min. By contrast, exhaustive enumeration of all combinations among 92 candidates would require $C_{92}^{N}$ separate FBA solves. Using the COBRA Toolbox’ s optimizeCbModel (average 0.0605 s per FBA, SD 0.0242 s), we estimate that full enumeration would take from ~4,200 solves for *N* = 2 up to ∼9.3e+10 solves for *N* = 8, making exhaustive search strictly infeasible (Fig. S3).

### Model preprocessing

*E. coli:* A methanol assimilation pathway consisting of 3 heterogeneous reactions encoded by RuMP methanol assimilation passway (*medh*: methanol dehydrogenase, *hps*: 3-hexulose-6-phosphate synthase, and *phi*: 3-hexulose-6-phosphate isomerase) was added to the *E. coli* model iML1515 [37]. Two reactions, “PFL” catalyzed by pyruvate formate lyase, and “OBTFL” catalyzed by 2-oxobutanoate formate lyase, were deactivated in the model as they are only active under anaerobic conditions. Additionally, the reactions “DRPA” and “PAI2T” were also removed since they are reported unrealistic [38].

*C. glutamicum*: The genome scale metabolic model iCW773 [39,40] for *C. glutamicum* ATCC 13032 was utilized, and methanol assimilation pathways (*medh*, *hps*, and *phi*) were integrated into the model. To enable xylose utilization, a xylose transport reaction was also added.

For all models, nonessential carbon fixation reactions were deactivated except for those catalyzed by phosphoenolpyruvate carboxylase, carbamate kinase, isocitrate dehydrogenase, carbamoyl-phosphate synthase, pyruvate carboxylase, acetyl-CoA carboxylase, and methylmalonate-semialdehyde dehydrogenase.

### Evaluation metrics of the metabolic engineering strategies for the starting strains for ALE

AdaptUC offers different metabolic engineering strategies for specific hosts and substrates. However, selecting the most effective strategies for designing the initial strain for ALE requires careful assessment. For a successful cosubstrate-aided ALE, the strain at the beginning of the ALE process must be able to grow on media containing the unadapted carbon source. Since it is unlikely for a starting strain to quickly uptake the unadapted carbon source, a low requirement for the unadapted source is crucial for survival. We quantify this with the parsimonious unadapted carbon source and cosubstrate assimilation ratio (UC/Co), which is the minimum uptake rate of an unadapted carbon source relative to the minimum uptake rate of a cosubstrate. A lower UC/Co indicates that a less unadapted carbon source is required for the survival of the starting strain. To obtain UC/Co, we set a minimum specific growth rate of 0.1 h^−1^ as a constraint for FBA to determine the minimum unadapted carbon source uptake rate. Subsequently, by fixing this minimum unadapted carbon source uptake rate, we determine the minimum cosubstrate uptake rate. The ratio of these 2 uptake rates defines UC/Co.

Equally importantly, there must be sufficient evolutionary drive for the strain to utilize the unadapted substrate as the sole carbon source. This represents the selective pressure exerted on the microbial population to adapt and acquire traits necessary for utilizing previously unadapted carbon sources for growth. The response to selective pressure is described by the following equation: [36, 37]

$$∆\bar{z}=GP^{-1}s$$ (35)

where s is the selection differential (the growth rate difference between evolved and unevolved strains, Δμ), $∆\bar{z}$ denotes the change of mean traits (the change in uptake rate of the unadapted carbon source, $\Delta v_{\mathrm{EX}}$), and *G* and *P* are the matrices of genetic and phenotypic variance–covariance, respectively [41,42]. The relative mean fitness gain from assimilating the unadapted carbon source can be expressed as:

$$\text{SADF}=G^{-1}P=\frac{s}{∆\bar{z}}=\frac{\Delta \mu }{\Delta v_{\mathrm{EX}}}$$ (36)

We define this relative mean fitness gain as SADF. A higher SADF indicates greater selective pressure and a higher feasibility of evolution. To encompass the evolutionary driving force across various unadapted carbon source uptake rates, we compute the average SADF. This calculation involves determining the growth difference between the genetically engineered initial strain on a sole cosubstrate ($v2^{\mathrm{bio}}$) and the evolved strains assumed to regain the growth rate achieved by the initial strain on a cosubstrate ($v0^{\mathrm{bio}\_\text{cosub}}$), through assimilating the unadapted carbon source from a mixed medium containing both unadapted and conventional carbon sources. The denominator is the minimal uptake rate of the unadapted carbon source $v^{\mathrm{UC}}$(calculated with Eqs. 23 to 28). The average SADF can be formulated as:

$$\bar{\text{SADF}}=\frac{v0^{\mathrm{bio}\_\text{cosub}}-v2^{\mathrm{bio}}}{v^{\mathrm{UC}}}$$ (37)

Unlike conventional objective functions that directly optimize fluxes (e.g., maximizing biomass or substrate uptake), SADF is a ratio-based descriptor. It quantifies the gain in growth per additional unit of previously unadapted substrate consumed. This distinction makes SADF particularly suitable for modeling ALE, since it explicitly measures the evolutionary pressure to couple growth with assimilation of a novel substrate.

## Data Availability

The authors declare that the data supporting the findings of this study are available within the paper and its Supplementary Materials. The main AdaptUC source code can be downloaded from Github (https://github.com/Jingyi-Cai/AdaptUC).
